# T Cell Defects: New Insights Into the Primary Resistance Factor to CD19/CD22 Cocktail CAR T-Cell Immunotherapy in Diffuse Large B-Cell Lymphoma

**DOI:** 10.3389/fimmu.2022.873789

**Published:** 2022-04-27

**Authors:** Jiachen Wang, Kefeng Shen, Wei Mu, Weigang Li, Meilan Zhang, Wei Zhang, Zhe Li, Tong Ge, Zhoujie Zhu, Shangkun Zhang, Caixia Chen, Shugang Xing, Li Zhu, Liting Chen, Na Wang, Liang Huang, Dengju Li, Min Xiao, Jianfeng Zhou

**Affiliations:** ^1^Department of Hematology, Tongji Hospital, Tongji Medical College, Huazhong University of Science and Technology, Wuhan, China; ^2^Immunotherapy Research Center for Hematologic Diseases of Hubei Province, Wuhan, China; ^3^Department of Pediatric Surgery, Tongji Hospital, Tongji Medical College, Huazhong University of Science and Technology, Wuhan, China; ^4^Perfectgen Diagnostics, Ezhou, China; ^5^Wuhan Bio-Raid Biotechnology Co., Ltd., Wuhan, China

**Keywords:** CAR-T cell immunotherapy, immune resistance, primary immunodeficiencies, T cell dysfunction, germline alterations, LDH – lactate dehydrogenase, cytokine release syndrome (CRS), DLBCL - diffuse large B cell lymphoma

## Abstract

Despite impressive progress, a significant portion of patients still experience primary or secondary resistance to chimeric antigen receptor (CAR) T-cell immunotherapy for relapsed/refractory diffuse large B-cell lymphoma (r/r DLBCL). The mechanism of primary resistance involves T-cell extrinsic and intrinsic dysfunction. In the present study, a total of 135 patients of DLBCL treated with murine CD19/CD22 cocktail CAR T-therapy were assessed retrospectively. Based on four criteria (maximal expansion of the transgene/CAR-positive T-cell levels post-infusion [C_max_], initial persistence of the transgene by the CAR transgene level at +3 months [T_last_], CD19+ B-cell levels [B-cell recovery], and the initial response to CAR T-cell therapy), 48 patients were included in the research and divided into two groups (a T-normal group [n=22] and a T-defect [n=26] group). According to univariate and multivariate regression analyses, higher lactate dehydrogenase (LDH) levels before leukapheresis (hazard ratio (HR) = 1.922; *p* = 0.045) and lower cytokine release syndrome (CRS) grade after CAR T-cell infusion (HR = 0.150; *p* = 0.026) were independent risk factors of T-cell dysfunction. Moreover, using whole-exon sequencing, we found that germline variants in 47 genes were significantly enriched in the T-defect group compared to the T-normal group (96% vs. 41%; p<0.0001), these genes consisted of CAR structure genes (n=3), T-cell signal 1 to signal 3 genes (n=13), T cell immune regulation- and checkpoint-related genes (n=9), cytokine- and chemokine-related genes (n=13), and T-cell metabolism-related genes (n=9). Heterozygous germline *UNC13D* mutations had the highest intergroup differences (26.9% vs. 0%; *p*=0.008). Compound heterozygous *CX3CR1*^I249/M280^ variants, referred to as pathogenic and risk factors according to the ClinVar database, were enriched in the T-defect group (3 of 26). In summary, the clinical characteristics and T-cell immunodeficiency genetic features may help explain the underlying mechanism of treatment primary resistance and provide novel insights into CAR T-cell immunotherapy.

## Introduction

CAR T- cell immunotherapy has demonstrated unprecedented efficacy in relapsed/refractory large B-cell lymphoma (1). Previously, we reported the remarkable safety and efficacy of CD19/22 CAR T-cell cocktail immunotherapy alone and following autologous stem cell transplantation (ASCT) in the treatment of adult patients with r/r B-cell malignancies (2–7). However, a substantial number of patients treated with CAR T cells may experience primary (no response, NR) or secondary (initial response followed by relapse/escape) resistance.

Primary resistance occurs at significantly higher rates in diffuse large B-cell lymphoma (DLBCL) (27% to 48%) than in B-cell precursor acute lymphoblastic leukemia (B-ALL) (19%), follicular lymphoma (14%), and mantle cell lymphoma (16%) with tisagenlecleucel and lisocabtagene maraleucel (8). Several studies reported that primary resistance was correlated with weaker expansion (maximal expansion of transgene/CAR-positive T-cell levels post-infusion [C_max_]) and shorter persistence (CAR transgene level at +3 months [T_last_]) of CAR T cells in r/r non-Hodgkin lymphoma (NHL) (9–11). In addition, the potent antitumor activity of CD19 CAR T cells in patients is associated with long-term B-cell aplasia (BCA) (12). In this study, T-cell dysfunction-related primary resistance was assessed by the expansion (C_max_) and the persistence (T_last_) of the CAR transgene, CD19^+^B cell aplasia, and initial response after CAR T-cell infusion. In contrast, the mechanisms of T-cell dysfunction-related primary resistance remain poorly understood, in which extrinsic and intrinsic factors might play roles.

Extrinsic factors might influence CAR T-cell function. It has been reported that an immunosuppressive tumor microenvironment (TME), such as CD4^+^CD25^+^ regulatory T cells and myeloid-derived suppressor cells and their respective proinflammatory factors, may generate resistance to CAR T cell treatment (13). Disease burden can positively affect the degree of cell expansion in B-ALL, which in turn might increase the risk and severity of cytokine release syndrome (CRS) (14, 15). In addition, a high tumor burden might trigger an aberrant immune microenvironment and T cell exhaustion (16). However, Liu et al. reported that no explicit significance was found in the relationship between tumor burden and CAR T-cell expansions and persistence in r/r DLBCL (17). Other risk factors, including cytokines, inhibitory receptors, and competition for nutrients within the TME, also contribute to CAR T cell dysfunction (18). Moreover, the influence of meditation, such as corticosteroids, tocilizumab, and bendamustine, is still controversial and needs to be further studied.

T-cell dysfunction can also be driven by T cell-intrinsic factors. The relevant studies have focused on three fields. First, the inherent T cell memory phenotype abnormalities revealed by flow cytometry showed that an elevated frequency of CD27^+^CD45RO^–^CD8^+^ T cells was associated with sustained remission (11). Second, characteristic transcriptomic profiling indicated by RNA sequencing showed that T cell clusters with higher expression of cytotoxicity (PRF1, GZMB, and GZMK) and proliferation genes were corrected with good ability in expansion and persistence (19). Third, next-generation sequencing (NGS) studies suggested that transgenes integrated into the *TET2* locus may also occur in CAR T-cell therapy (20). In addition to these alterations, inborn errors of immunity, referred to as primary immunodeficiencies (PIDs), also participate in the mechanism of intrinsic T-cell defects. PID is caused by monogenic germline mutations that result in loss of function (hypomorphic), or gain-of-function (hypermorphic) of encoded protein (21). Currently studies on germline alterations are limited in CAR T-cell immunotherapy.

Germline genetic aberrations may have indications for targeted agents. For example, in the field of targeted immunotherapy, microsatellite instability and mismatch repair deficiency, which may arise from *MLH1*, *MSH2*, *MSH6*, and *PMS2* mutations, suggests potential vulnerability to PD-1 inhibitors (22). Olaparib, a poly polymerase inhibitor, is approved as maintenance therapy for patients with advanced pancreatic cancer and a germline *BRCA1* or *BRCA2* pathogenic ovarian cancer (23). Genetic studies of DLBCLs in humans have revealed an increasing number of potentially relevant germline alterations (24). However, in the field of CAR T-cell immunotherapy, it remains unclear whether germline mutations affect cellular kinetic T-cell function. T cell germline defects add another layer of complexity in understanding the CAR T-cell therapy resistance mechanism and provide novel insight into targeted drug developments.

In this study, we analyzed the clinical and genetic characteristics of 48 r/r DLBCL patients receiving CD19/CD22 cocktail CAR T-cell therapy, aiming to characterize the prognostic factors of T cell dysfunction related to the primary resistance mechanism. This work may help explain the underlying mechanisms of primary resistance to treatment and provide novel insights into CAR T-cell immunotherapy.

## Materials and Methods

### Patient Population

In our study, patients with DLBCL treated with murine CAR T-cell cocktail therapy at Tongji Hospital between January 2019 and August 2020 were enrolled according to a previous report (2, 7). Two clinical trials (Trial A and Trial B) were included in the analysis. Trial A involves a murine CAR19/22 T-cell “cocktail” therapy, and Trial B involves an ASCT followed by CAR19/22 T-cell “cocktail” therapy. The timeline of leukapheresis, leukodepletion, chimeric antigen receptor therapy T-cell (CAR-T) infusion, and the follow-up period are described in **Figure S1**.

**Figure 1:**
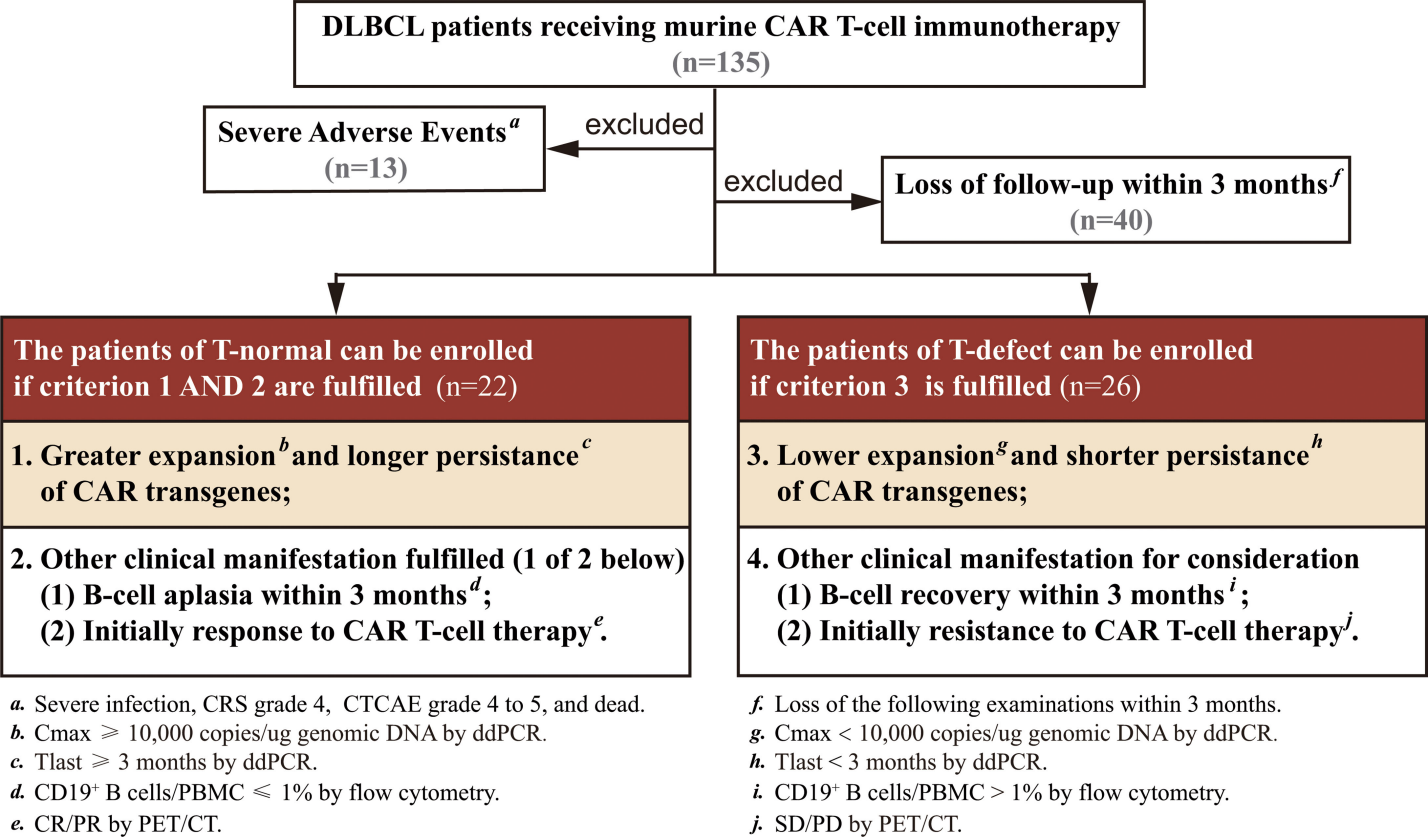
Flow diagram summarizing patient recruitment, exclusion criteria, and patient groups. Patients were divided into a T-normal group (n=22) and T-defect group (n=26) according to the criteria of CAR transgene expansion, persistence, BCA, and initial response to CAR T-cell therapy. BCA, B-cell aplasia; CR, complete remission; CRS, cytokine release syndrome; CTCAE, common terminology criteria adverse events; ddPCR, droplet digital PCR; PET/CT, positron emission tomography-computed tomography; PR, partial remission; SD, stable disease; PD, disease progression.

All the patients were followed up until they died, lost to follow-up, or withdrew consent. A series of screening conditions were set up to select patients with typical T-cell characteristics (**Figure 1**). Patients were divided into 2 groups: a T-normal group (n=22) and a T-defect group (n=26). Grouping was based on four criteria (maximal expansion of transgene/CAR-positive T-cell levels post-infusion [C_max_], initial persistence of transgene by CAR transgene level at +3 months [T_last_], CD19^+^ B-cell levels [B-cell recovery], and initial response assessment after CAR-T cell infusion). Patient characteristics and outcomes were collected retrospectively. The raw data are shown in **Table S1**.

Further details regarding the study procedures are described in the **Supplementary Methods**. This study was carried out following the Declaration of Helsinki and approved by the Medical Ethics Committee of the Department of Hematology, Tongji Hospital, Tongji Medical College, Huazhong University of Science and Technology (ChiCTR-OPN-16009847, ChiCTR-OPN-16008526). Because of the retrospective nature of the study and that the specimens used were the remaining samples of clinical testing retrospectively, free of additional harm to the patients, the need for informed consent was waived.

### Bioanalytical Methods

Peripheral blood was collected from patients to evaluate post-infusion CAR transgene levels *via* droplet digital PCR (ddPCR). The details related to the analytical methods have been previously published (2). CAR transgene was detected by ddPCR measurements before lymphodepletion chemotherapy; just after infusion; on days 4, 7, 11, 14, 17, 21, and 28; and at months 2 and 3. Bone marrow collection occurred at screening, day 28 if the patient was in complete response (CR), and at month 3. Partitioning of the CAR transgene was assessed by the ratio of bone marrow concentrations to peripheral blood levels. Cellular kinetics exposure parameters included maximal expansion of transgene/CAR-positive T-cell levels post-infusion (C_max_) and persistence (duration transgene/CAR-T cells are present in peripheral blood and tissues [T_last_]). The results are reported as transgene copies/micrograms of genomic DNA for ddPCR. CD19^+^ B-cell levels were evaluated pre-/post-infusion to monitor B-cell aplasia *via* flow cytometry (25).

Staging and response assessments were defined according to the National Comprehensive Cancer Network guidelines and Lugano Treatment Response Criteria (26). CRS was graded according to the scale proposed by Lee et al. (27). Risk factors for the CRS grade included high marrow tumor burden, lymphodepletion *via* cyclophosphamide and fludarabine, higher CAR-T cell dose, thrombocytopenia before lymphodepletion, and manufacturing of CAR-T cells without selection of CD8^+^ central memory T cells (28). CAR T cell-related encephalopathy syndrome and other adverse events (AEs) were evaluated according to Common Terminology Criteria for Adverse Events (CTCAE) v.4.03 (29).

Tumor burden was approximated using lactate dehydrogenase (LDH) levels before leukapheresis or CAR-T cell infusion, and maximum tumor diameter (MTD) was measured on CT or positron emission tomography/computed tomography (PET/CT) scans (30). Interphase fluorescence *in situ* hybridization (FISH) was performed using commercially available probes (Abbott Molecular, Downers, Grove, IL, USA). LSI IGH/IGHV (14q32), LSI MYC (8q24) Dual Color, break-apart rearrangement probes were used to detect the rearrangement of *BCL2*, *BCL6*, and *C-MYC*, respectively. A 17p13.1 (P53) probe (Vysis, Downers, Grove, IL) was used to detect 17p deletions. Sample preparations and hybridizations were conducted following the manufacturer’s recommendations, and 200 cells were analyzed for each probe as previously reported (31).

### Targeted Sequencing Analysis

Targeted high-throughput sequencing was applied for somatic alterations. A total of 57 genes were selected in this study (listed in **Table S2**). Most genes were frequently altered in DLBCL, according to data from several previously published large-scale DLBCL group studies (32–34). Using genome build hg19/GRCh37 as a reference, a sequencing panel covering the coding sequences within five intronic base pairs around exons in 57 genes was designed online (Designstudio Sequencing, Illumina, San Diego, USA). Sequencing libraries were prepared with AmpliSeq™ Library PLUS for Illumina, using 20 ng of input genomic DNA per sample. Library sequencing was performed to 2000X coverage on a NextSeq™ 550 system using an Illumina NextSeq™ 500/550 High Output v2 Kit (Illumina, San Diego, USA). The alignment and variant calling were performed using a DNA Amplicon workflow with default parameters on BaseSpace Sequence Hub (Illumina). The generated variants were further annotated using ANNOVAR (35). Further details are described in the **Supplementary Methods**.

### Whole-Exome Sequencing (WES) Analysis

We performed WES for germline alteration analysis. The T cell-related gene panel included ten categories of CAR-T and T-cell biology (I=CAR structure; II=TCR signal; III=T cell co-stimulation signal; IV= interleukin 2 (IL-2) signal; V=Immune dysregulation; VI=JAK-STAT signal; VII=Immune checkpoints; VIII=cytokines; IX=chemokines; and X=Metabolism). A total of 124 genes were enrolled in the panel (listed in **Table S3**). In addition, the gene panel of Human Inborn Errors of Immunity was set up according to the 2019 update on the classification from the International Union of Immunological Societies (IUIS) Expert Committee, the gene number of which was 403 (listed in **Table S4**) (21). Forty-nine genes (e.g., *CD19*, *CD3D*, *TNFRSF9*, *UNC13D*, *JAK3*, *IFNAR1*, *CSF3R*, *IL10*) overlapped in the two panels.

Genomic DNA was extracted from PBMCs with a QIAmp DNA Blood Mini kit (Qiagen, Germany) according to the manufacturer’s instructions. An Agilent SureSelect Human All ExonV6 Kit (Agilent Technologies, Santa Clara, CA, USA) was used for exome capture. The genomic DNA was sequenced by Illumina NovaSeq following the manufacturer’s protocols. BWA software aligned the raw data to the human genome (hg37). Public databases (1000G_EAS, ExAC_EAS, and GenomAD_EAS) were used to filter and remove common single-nucleotide polymorphisms (SNPs). Rare variants with minor allele frequency (MAF) ≤ 0.03 were included. The study strategies of germline and somatic mutations are displayed in **Figure S2**. Further details regarding the study procedures are described in the **Supplementary Methods**.

### Statistical Analysis

Patients’ baseline and clinical characteristics were described in **Tables 1**, **2**, using the means ± standard deviations for normally distributed continuous variables (e.g., age), medians and interquartile ranges (IQRs) for nonnormally distributed continuous variables (e.g., lines prior to CAR-T, cycles prior to CAR-T), and counts and percentages for categorical variables (e.g., male sex, disease stage). Student’s t test, the Mann-Whitney U test, and Pearson’s Chi-Squared test were applied to compare the above results. After assigned values for statistically significant variables (**Table 3**), the values included in the regression model were: score of maximal tumor diameter (MTD), score of LDH/upper limit of normal level before leukapheresis, score of CRS grade. Univariate and multivariate forward stepwise regression analyses were performed to identify the significant risk factors for the T-cell dysfunction related to primary resistance in **Table 4**. Statistical analysis was performed using GraphPad Prism 8 and SPSS version 19 software. *P* < 0.05 (2-sided) was considered statistically significant.

**Table 1:** Baseline characteristics of patients with and without T-cell defects after CAR-T therapy.

	All Patients (n=48)	T-normal (n=22)	T-defect (n=26)	*P*
**Age in years**	46.40 ± 11.52	49.52 ± 13.08	42.95 ± 8.57	0.081
**Male sex**	27 (56.3%)	12 (54.4%)	15 (57.7%)	0.827
**IPI score**				0.978
< 2 risk factors	13 (27.1%)	6 (27.3%)	7 (26.9%)	
≥ 2 risk factors	35 (72.9%)	16 (72.7%)	19 (73.1%)	
**Disease stage**				0.861
Stages I and II	4 (8.3%)	2 (9.1%)	2 (7.7%)	
Stages III and IV	44 (91.7%)	20 (90.9%)	24 (92.3%)	
**B symptom**				0.312
Yes	10 (20.8%)	6 (27.3%)	4 (15.4%)	
No	38 (79.2%)	16 (72.7%)	22 (84.6%)	
**Cell of origin of cancer**				0.165
Germinal center B-cell type	15 (31.3%)	10 (45.5%)	5 (19.2%)	
Nongerminal center B-cell type	25 (52.1%)	11 (50.0%)	14 (53.8%)	
Missing	8 (16.7%)	1 (4.5%)	7 (26.9%)	
**Lines prior to CAR-T**	3 (3-4)	3 (3-4)	3 (2-4)	0.815
**Cycles prior to CAR-T**	8 (7-11)	8 (7.5-11)	7 (6-12)	0.354
**CAR T-cell infusion regimen**				0.422
Murine CAR-T	21 (43.7%)	11 (50.0%)	10 (38.5%)	
Murine CAR-T following auto-HSCT	27 (56.3%)	11 (50.0%)	16 (61.5%)	
**Average dose of CAR T cells**				0.675
≤ 4 x 10^6^/kg	29 (60.4%)	14 (63.6%)	15 (57.7%)	
> 4 x 10^6^/kg	19 (39.6%)	8 (36.4%)	11 (42.3%)	

The values are presented as the means ± standard deviations or counts (percentages). IPI, International Prognostic Index; auto-HSCT, autologous hematopoietic stem cell transplantation.

**Table 2:** Univariate analysis of outcomes in patients treated with CAR-T cells.

	All Patients (n=48)	T-normal (n=22)	T-defect (n=26)	*P*
**Characteristics of patients**				
**HBV/HCV infection**	18 (37.5%)	10 (45.5%)	8 (30.8%)	0.295
**Bone marrow involvement**	14 (29.8%)	4 (18.2%)	10 (40.0%)	0.103
***P53* deletion detected by FISH**	14 (46.7%)	7 (50.0%)	7 (43.8%)	0.703
**Double-hit/triple-hit lymphoma**	4 (21.1%)	0 (0.0%)	4 (21.1%)	0.164
***TP53* mutation**	15 (38.5%)	5 (26.3%)	10 (50.0%)	0.129
**Tumor maximum diameter (cm)**	4.30 (2.10-6.75)	3.40 (1.65-4.50)	4.90 (4.10-9.20)	**0.010**
**Bendamustine**	2 (4.2%)	0 (0.0%)	2 (7.7%)	0.189
**Leukapheresis related factors**				
** Days from initial diagnosis to leukapheresis**	456.0 (295.5-770.75)	537.0 (327.5-827.0)	400.0 (221.0-683.0)	0.175
** LDH level before leukapheresis**	240.50 (198.5-787.3)	222.0 (190.0-329.5)	307.0 (210.00-559.0)	**0.020**
** Platinum-based drugs (3 months)**	22 (45.8%)	10 (45.5%)	11 (42.3%)	0.827
** CTX (3 months)**	15 (31.3%)	5 (22.7%)	10 (38.4%)	0.241
** Lenalidomide (3 months)**	3 (6.3%)	2 (9.1%)	1 (3.8%)	0.454
**CAR T-cell infusion-related factors**				
** Days from initial diagnosis to CAR T-cell infusion**	463.0 (298.0-787.3)	554.0 (309.5-841.0)	420.0 (236.0-694.0)	0.247
** LDH level before CAR T-cell infusion (IU/L)**	356.0 (195.8-414.8)	244.0 (186.0-557.5)	472.0 (255.0-685.0)	0.153
** Maximum LDH level prior to CAR T-cell infusion (IU/L)**	520.0 (274.5-1208.0)	365.0 (243.0-1012.5)	795.0 (414.0-1590.0)	0.102
** CRS**				**0.013**
Grade 0	14.9 (29.2%)	2 (9.1%)	12 (46.2%)	
Grade 1	19 (41.3%)	9 (40.9%)	10 (38.5%)	
Grade 2	13 (28.3%)	9 (40.9%)	4 (15.4%)	
Grade 3	2 (4.3%)	2 (9.1%)	0 (0.0%)	
Grade 4	0 (0.0%)	0 (0.0%)	0 (0.0%)	
**Dexamethasone**	12 (25%)	6 (27.3%)	6 (23.1%)	0.738

Values in bold refer to P-value <0.05. CRS, cytokine release syndrome; CTX, cyclophosphamide; FISH, fluorescence in situ hybridization; HBV, hepatitis B virus; HCV, hepatitis C virus; LDH, lactate dehydrogenase.

**Table 3:** Scores of factors that are significant in the univariate analysis.

Variable	Value	Score
**MTD (cm)**	<3	0
	3-5	1
	5-7.5	2
	7.5-10	3
	≥10	4
**LDH level before leukapheresis**	N times higher than ULN	N
**CRS grade**	0	0
	1, 2	1
	≥3	2

CRS, cytokine release syndrome; LDH, lactate dehydrogenase; MTD, maximal tumor diameter; ULN, upper limit of normal.

**Table 4:** Univariate and multivariate forward stepwise regression analysis.

Variable	Univariate analysis		Multivariable analysis		
	HR (95% CI)	P- Value	HR (95% CI)		P-Value
***Score of* MTD**	1.878 (1.117-3.159)	**0.017**	1.346 (0.737-2.456)		0.334
***Score of* LDH/ULN prior to leukapheresis**	2.141 (1.224-3.744)	**0.008**	1.922 (1.015-3.641)		**0.045**
***Score of*** **CRS grade**	0.113 (0.023-0.555)	**0.007**	0.150 (0.028-0.795)		**0.026**

Values in bold refer to P-value <0.05. 95% CI, 95% confidence interval; CRS, cytokine release syndrome; HR, hazard ratio; LDH, lactate dehydrogenase; MTD, maximal tumor diameter; ULN, upper limit of normal.

## Results

### Baseline Characteristics

From January 2019 to August 2020, 135 patients with r/r DLBCL were screened for eligibility, and all received murine CD19/CD22 CAR T-cell cocktail therapy. Forty-eight patients were retrospectively enrolled in the present study (**Figure 1**): 21 patients who received CAR T-cell infusion and 27 patients who received CAR T-cell therapy following ASCT. The detailed timeline and process of CAR T-cell infusion are shown in **Figure S1**.

The baseline characteristics are summarized in **Tables 1** and **S1**. There was no significant difference in age (median 49 vs. 43 years; *p*=0.081), international prognostic index (IPI) score (≥2 risk factors: 72.7% vs. 73.1%; *p*=0.978), disease stage (stage II, IV: 90.9% vs. 92.3%, *p*=0.312), or cell of origin (COO) (germinal center B-cell type: 45.5% vs. 19.2%, *p*=0.165) (36). In addition, the data in the two groups for lines (median 3 vs. 3; *p*=0.815) and cycles (median 8 vs. 7; *p*=0.815) prior to CAR-T were not significantly different. Besides, there was no difference in bridging treatment between two groups (ASCT: 50.0% vs. 61.50%; *p*=0.422). Furthermore, the average dose of CAR-T cells (>4 x 10^6^/kg: 36.4% vs. 42.3%; *p*=0.675) also did not significantly differ.

### T-Cell Functionality-Related Characteristics

**Figure 2:**
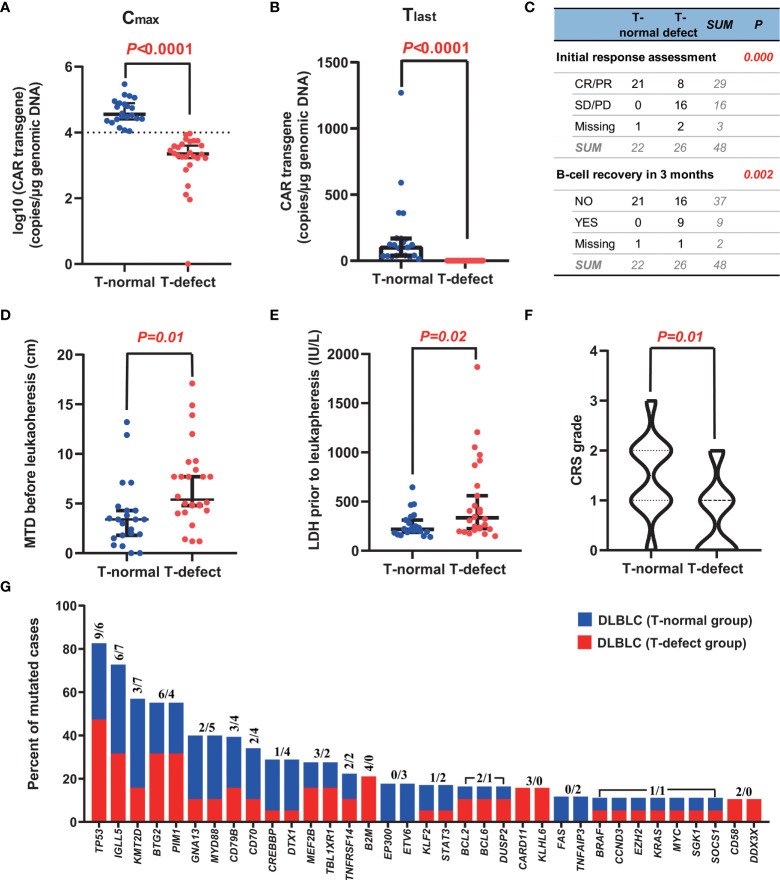
Typical characteristics of the two groups. **(A, B)** CAR T-cell expansion (C_max_) and persistence (T_last_ at +3 months) in peripheral blood were greater in the T-normal group than in the T-defect group (p<0.0001). **(C)** The initial response to CAR-T cell therapy was also considerably better in patients with T-normal function than in those with T-defect function (p<0.0001). In addition, there were significant differences in B-cell recovery in the T-normal group compared with the T-defect group (p=0.002). **(D, E)** MTD and LDH level in the T-normal and T-defect groups before leukapheresis demonstrate significant differences (p=0.01, and 0.02, respectively) according to the Mann-Whitney Test. **(F)** The T-normal group showed higher CRS grades than the T-defect group according to a Pearson chi-square test (p=0.01). **(G)** Recurrent somatic mutations in DLBCL. Shown is the prevalence of the indicated genetic abnormalities in 57 genes in the T-normal group (in blue) and T-defect group (in red). The two numbers for each mutation represent the counts of individuals carrying the genetic alterations in the T-defect and T-normal groups, respectively. The somatic origin of the mutations was confirmed by analysis of paired PBMC germline DNA. CAR, chimeric antigen receptor; CR, complete remission; CRS, cytokine release syndrome; PD, disease progression; PR, partial remission; SD, stable disease; SNP, single nucleotide polymorphism; MTD, maximal tumor diameter.

Four T-cell functionality-related primary resistance factors were analyzed between the two groups. The C_max_ of CAR transgene DNA (*p*<0.0001) and T_last_ of transgene level at three months (*p*<0.0001) were significantly lower in the T-defect group than in the T-normal group (**Figures 2A, B**). Within three months after CAR T-cell infusion, B-cell recovery rates differed considerably between the two groups (0% in the T-normal; 37.5% in the T-defect; *p=0.002*) (**Figure 2C**). Moreover, the T-defect group had a lower response (CR/PR at initial assessment after CAR T-cell infusion) rate (33.3% vs. 100%, *p<0.0001*) than the T-normal group did (**Figure 2C**). In summary, T-cell functionality differed markedly between the two groups, which was the basis for subsequent statistical analysis.

### Univariate Analysis

Factors related to disease characteristics, leukapheresis, and CAR T-cell infusion were explored (**Table 2**). The *p53* deletion incidence was 50.0% in the T-normal group and 43.8% in the T-defect group (*p*=0.703). Although not statistically significant, the bone marrow infection rate (40.0% vs. 18.2%; *p*=0.103), double hit/triple-hit lymphoma incidence (21.1% vs. 0%; *p*=0.163), *TP53* mutation rates (50% vs. 26.3%; p= 0.129), and bendamustine usage before leukapheresis (7.7% vs. 0%; p=0.189) were higher in the T-defect group than in the T-normal group. However, there was no significant difference in platinum-based, cyclophosphamide, or lenalidomide drug use within three months before leukapheresis (*p*>0.05). The median value of MTD (4.90 vs. 3.40; *p*=0.010; **Figure 2D**) and the LDH level before leukapheresis (307.0 vs. 222.0; *p*=0.020; **Figure 2E**) were higher in the T-defect group than in the T-normal group. In contrast, instant LDH (median: 472.0 vs. 244.0; *p*=0.153) and maximum LDH levels (median: 795.0 vs. 365.0; *p*=0.102) before CAR T-cell infusion were not significantly different between the two groups. Moreover, the CRS grade was significantly lower in the T-defect group (*p*=0.013) (**Figure 2F**).

### Univariate and Multivariate Logistic Regression Analysis

Uni- and multivariate logistic regression analyses of overall survival (OS) were performed by including broad groupings of patient characteristics to define the clinical factors correlated with T-cell dysfunction. The three risk factors above, namely, MTD, LDH level before leukapheresis, and CRS grade after CAR T-cell infusion, were included in the regression analysis. First, values were assigned for these variables, as listed in **Table 3**. Second, univariate logistic regression analysis revealed that these factors were statistically significant risks (**Table 4**). Furthermore, in the multivariable regression analysis, compared to T-normal group, patients with T-cell dysfunction (T-defect group) were associated with a significantly higher risk of LDH/ULN prior to leukapheresis (hazard ratio (HR) =1.922, 95% confidence interval (95% CI) 1.015-3.641, *p*=0.045) and decreased risk of CRS grade (HR=0.150, 95%CI 0.028-0.795, *p*=0.026) but no increased risk in MTD (HR=1.346; 95% CI=0.737-2.456; *p*=0.334). In summary, LDH/ULN before leukapheresis was associated with a significantly higher risk of T-cell dysfunction, and CRS grade was the only independent favorable factor.

### Somatic Features of the Genetics of the Two Groups

Targeted NGS was performed to investigate the somatic genetic alterations. Among the 48 patients, 36 samples were obtained from initial diagnosed formalin-fixed paraffin-embedded tissue (n=29) or peripheral blood circulating tumor DNA (n=7), and performed targeted NGS. A total of 259 somatic mutations (MAF ≤ 0.01) in 57 genes were identified, namely, 13 splice-site mutations, 176 missense mutations, 24 truncated mutations, 27 frameshift insertions/deletions, and 19 non-frameshift insertions/deletions (**Table S5**), exclusively in tumor cells compared to peripheral blood mononuclear cells (PBMCs). Forty-seven mutated genes were detected in the 36 “screened” cases. The most frequently mutated genes included the tumor suppressor factor gene *TP53* (42%, 16 of 36), immunoglobulin variable gene *IGLL5* (36%, 13 of 36), and epigenetic regulator gene *KMT2D* (28%, 10 of 36) (37). There was no significant difference in somatic mutations between the two groups (*p*>0.05) (**Figure 2G**). Somatic clonal evolution of three patients in the T-defect group (**Figure S3**).

### Germline Features of the Genetics of the Two Groups

**Figure 3:**
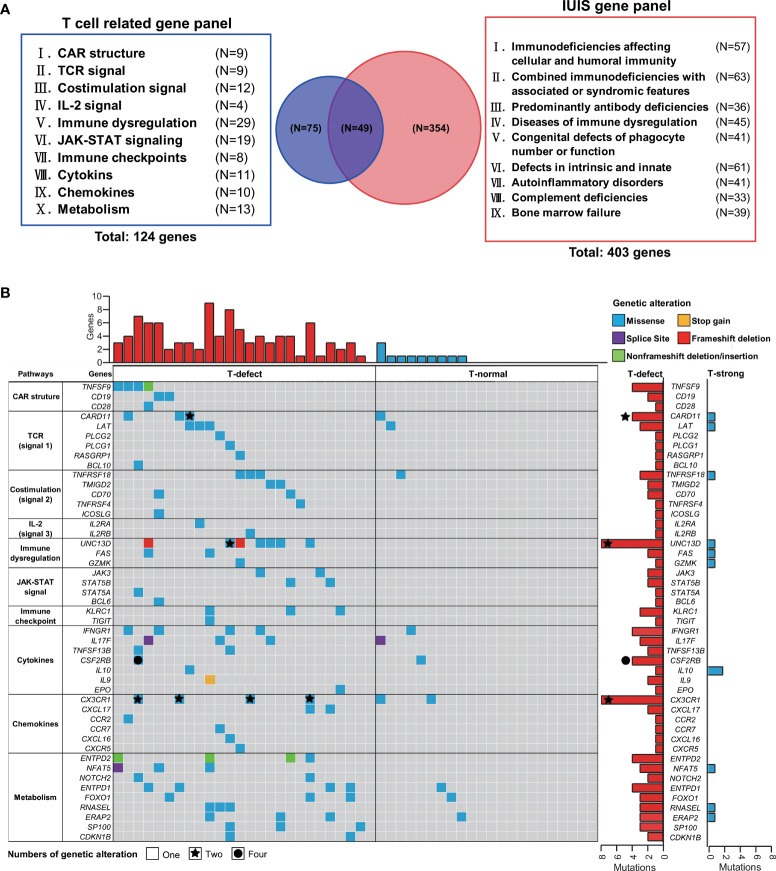
Targeted gene panel of T-cell functions and waterfall plot of germline mutations. **(A)** One hundred and twenty-six target genes, including ten T-cell and CAR-T cell biology categories, were selected for the waterfall plot with T-cell grading information. Fifty genes were identical to primary genetic defects reported by the IUIS/WHO committee. **(B)** The top 47 mutated genes that differed between the two groups, such as *TNFSF9*, *CD19*, *CARD11*, *UNC13D*, and *CX3CR1*, were selected for the waterfall plot with T-cell group information. The genes were arranged according to the T cell-related gene panel in **(A)**. Each column corresponds to a sample, and cases are ordered by the lymphoma with T-defect on the left (red bar) and with T-normal (blue bar) on the right. The types of genetic alterations are shown as different colors as shown in the legend in the upper-right corner. The counts of genetic alterations are shown as none, stars, and circles, representing once, twice, and four times person-times, respectively. CAR, chimeric antigen receptor; JAK-STAT, Janus kinase-signal transducer and activator of transcription; IL-2, interleukin-2; IUIS, International Union of Immunological Societies; TCR, T-cell receptor; WHO, World Health Organization.

**Figure 4:**
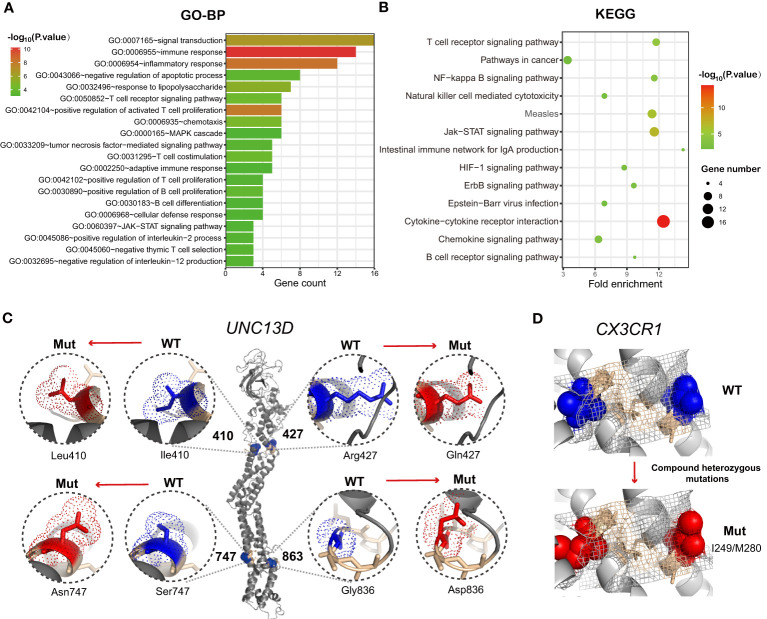
**(A)** Histograms showing the top 20 GO-BP enrichment results of 47 differentially mutated genes between the T-defect and T-normal groups in **Figure 3B**. The x-axis represents the enriched gene count, and the intensities of the different colors represent the p values. **(B)** Bubble diagram showing the top 13 KEGG enrichment items of differentially expressed genes between the patient and two healthy donors. The x-axis represents the gene ratio, and the intensities of the different colors represent the p-values. **(C, D)** *UNC13D* mutations and *CX3CR1* compound heterozygous mutations were the most frequent germline alterations in the patients. Shown is a ribbon cartoon indicating the locations of WT and mutants in the UNC13D and CX3CR1 proteins. The figures were prepared *via* PyMOL (www.pymol.org). Four *UNC13D* alterations are reported in ClinVar (rs766652119, rs117221419, rs140184929, rs9904366). Most variants in *UNC13D* were frameshift and missense variants. The UNC13D^p.Arg1077SerfsTer48^ variant [NM_199242.3(UNC13D):c.3229_3235del (p.Arg1077fs)] is defined as pathogenic by the American College of Medical Genetics and Genomics (ACMG) and is suspected for for pathogenicity for familial hemophagocytic lymphohistiocytosis (HLH). GO-BP, Gene Ontology-Biological Process; KEGG, Kyoto Encyclopedia of Genes and Genomes; WT, wild type; Mut, mutant.

The inherent T cell phenotype of CAR T cells can affect post-infusion CAR T-cell behavior (38). Intrinsic T-cell dysfunction was linked to inborn T cell biology-related genes (21). Therefore, WES of patient PBMCs was performed to explore germline genetic features. A T-cell-related gene panel containing 124 genes was constructed (**Figure 3A**). Patients in the T-defect group (counts average: 6; IQR: 4-8) harbored significantly more germline variants of the T-cell-related genes (counts average: 3; IQR: 2-6) than those in the T-normal group (**Table S6**). Forty-seven genes were presented in the factorized mutational heatmap by groups in the order of the T cell-related gene panel (**Figure 3A**) that met one of the following conditions: 1) the variants were presented only in the T-defect group, 2) the percentage in the T-defect group was more than two times than that in the T-normal group. The top 47 mutated genes that differed between the two groups were selected for the waterfall plot (96% vs. 41%; *p*<0.0001). Genes were arranged according to the order of the T cell-related gene panel (**Figure 3B**). The chi-square tests indicated that gene variants of CAR structure (*p*=0.016), T cell receptors (TCR) signaling (*p*=0.036), co-stimulation signaling (*p*=0.020), immune dysregulation (*p*=0.004), JAK/STAT signaling (*p*=0.016), chemokines (*p*=0.036), and T-cell metabolism (*p*=0.002) were higher in the T-defect group than in the T-normal group. The IL-2 signal (*p*=0.184), immune checkpoint (*p*=0.100), and cytokines (*p*=0.054) were not different between the two groups. Gene Ontology (GO) and Kyoto Encyclopedia of Genes and Genomes (KEGG) analyses of the 47 differentially expressed genes enriched in the T-defect group indicated enrichment in several T cell-related immunodeficiency pathways and JAK/STAT, NF-κB, and HIF-1 signaling pathways (**Figures 4A, B**).

Heterozygous germline *UNC13D* mutations presented the highest intergroup differences (26.9% vs. 0%; *p*=0.008). Six heterozygous mutants were found in *UNC13D*. P11 and P39 shared the same missense mutation [c.1228A>C(p.Ile410Leu)]. P31 and P38 shared another frameshift deletion [c.3229_3235del; p.Arg1077SerfsTer48]. **Figure 4C** shows the protein structure of wild-type (WT) and heterozygous mutants in *UNC13D* with PyMOL software, which included the following variants: c.1228A>C(p.Ile410Leu), c.1280G>A(p.Arg427Gln), c.2240G>A(p.Ser747Asn), and c.2588G>A(p.Gly863Asp). Except for the five variants below, P11 harbored a missense mutation [c.175G>A(p.Ala59Thr)] that was beyond the modeling scope of PyMOL software. Compound heterozygous *CX3CR1* variants [c.841G>A(p.Val281Ile), and c.935C>T(p.Thr312Met)], were enriched in the T-defect group (3 of 26). The ClinVar database indicated that these two compound heterozygous mutations were *CX3CR1* (dbSNP:rs3732378, and dbSNP:rs3732379, https://www.ncbi.nlm.nih.gov/clinvar/variation/8152/), whose clinical significance was defined as pathogenic to human immunodeficiency virus type 1 infection and as a risk factor for age-related macular degeneration 12. Variants of WT and *CX3CR1*^I249/M280^ structures were analyzed and displayed using PyMOL in **Figure 4D**.

## Discussion

**Figure 5:**
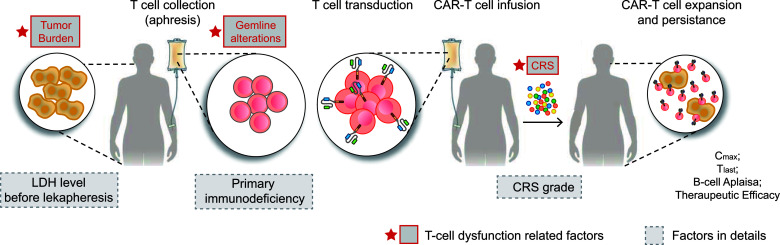
CAR-T cell therapy and T-cell dysfunction-related factors. CAR-T therapy involves separating a patient’s T cells *via* apheresis and then genetically engineering the cells to produce receptors on their surfaces, called CARs. CARs are fusion proteins of an antigen-recognition domain from a monoclonal antibody and one or more T-cell receptors. They allow T cells to recognize and attach to specific proteins, namely tumor antigens. T cells counts are expanded to hundreds of millions, after which the cells are then infused back into the patient, selectively destroying chemotherapy-resistant cancer cells. Patients receiving CAR-T are at risk for developing CRS, an inflammatory response that occurs secondary to cytokine release by infused CAR-T cells. CRS is characterized by fevers, hypotension, tachycardia, elevated inflammatory marker levels, and end-organ damage, including acute kidney injury and neurotoxicity. In summary, tumor burden (LDH level before leukapheresis), germline alterations (T cell-related PIDs), and CRS (CRS grade) were factors associated with CAR T-cell function. CAR, chimeric antigen receptor; CRS, cytokine release syndrome; LDH, lactate dehydrogenase.

The clinical characteristics and germline genetic framework for DLBCL that we present here provide a new and evolving understanding of the primary resistance of CAR T-cell immunotherapy and the molecular attributes that may influence therapeutic response. One key idea of this study is that T-cell dysfunction-related primary resistance could be measured by four parameters: CD19 CAR transgene expansion, persistence, CD19^+^ B cell recovery, and therapeutic response in CAR T-cell immunotherapy. Unlike previous investigations showing that T-cell dysfunction-related primary resistance to CART19 mainly focused on the T cell memory phenotype, exhausted transcriptomic profiling, and acquired T cell destruction (11, 19, 20, 39), our study revealed a novel model that contributed to weak CAR T-cell expansion and persistence. There are three overarching phases and implications of these findings as follows: an intrinsic resistance response to T-cell related heterozygous germline alterations (e.g., *UNC13D*, *CX3CR1* mutations), followed by an extrinsic high antigen-driven T cell dysfunction measured by higher LDH level before leukapheresis, finally with the manifestation of low CRS severity (**Figure 5**).

A higher LDH level before leukapheresis was an independent risk factor for T-cell dysfunction in this study. Elevated LDH at the time of pre-infusion or pre-lymphodepletion was associated with early therapeutic response, early relapse, shorter progression-free survival (PFS), and shorter OS in B-NHL patients receiving murine CD19 CAR T-cell therapies (40–43). The presence of high lactate levels in the TME is usually associated with an acidic extracellular pH (6.5) and a lower number and activity of CD8^+^ T cells and natural killer (NK) cells both *in vitro* and *in vivo*. High LDH levels have been shown to suppress T-cell functions, including IL-2 secretion and TCR activation (44). Together, these observations suggest that proceeding with leukapheresis earlier when the TMB is low in treatment may benefit patients more from CAR T-cell therapy.

CRS, the most common toxicity of cellular immunotherapy, is triggered by the activation of T cells upon the engagement of their TCRs or CARs with cognate antigens expressed by tumor cells (29). Expansion of the CAR transgene was associated with CRS severity in B-ALL and DLBCL, in accordance with our research (45). We suspect that CRS symptoms manifest T cell cytotoxicity *in vitro* and help doctors estimate patients’ T-cell function early and quickly. Since severe AEs were excluded from our study, the influence of life-threatening CRS on cellular kinetics warrants future research.

Pathogenic germline alterations provide evolving insights into primary resistance mechanisms. Previously, our therapeutic center reported two patients who harbored germline mutations and received murine monoclonal anti-CD19 and anti-CD22 CAR T-cell “cocktail” therapy (5, 46). A pathogenic *PIM1* mutation (c.403G>A, p.Glu135Lys, heterozygous) was detected in a MYC/BCL2/BCL6 triple-hit DLBCL patient, and a pathogenic *TP53* germline mutation (c.818G>A, p.R273H, heterozygous) was found in another DLBCL patient (5, 46). These two patients had weak C_max_ and T_last_ values (C_max_ < 10,000 copies/μg, T_last_ < 3 months), and the disease progressed, which met the criteria of “T-defect” group. So these two patients were suspected of having T-cell dysfunction in CAR T-cell immunotherapy. In the present study, the polygenic inheritance pattern may play a role in T-cell dysfunction.

Some germline variants are too damaging to be compatible with normal organism function, leading to monogenic inherence disease. In contrast, some germline variants may also remain asymptomatic or lead to milder disease. Compared with healthy people, patients who harbor germline mutations may be more prone to severe symptoms (47). A multistep pathogenesis for immune diseases has been suggested, in which multiple variants, both inherited and somatic ones, contribute to the emergence of disease (48). For example, secondary hemophagocytic lymphohistiocytosis (HLH) is a life-threatening hyperinflammatory disease that may have a polygenic inheritance model. Heterozygous variants in the various “polygenic” dual gene combinations were found in various analyses (49). Not surprisingly, the genes implicated in single-gene disorders have also been linked to polygenic disorders. Polygenic inheritance patterns are likely to account for more common systemic autoimmune diseases (50).

T cell biology- and CAR-T cell structure-relevant genes were included in our analysis (n=124). Interestingly, there was considerable overlap with PID genes (n=50). An effective T cell response requires both signal one (TCR/CD3-ζ) and signal two (costimulatory signals, such as CD28 or 4-1BB). In addition, IL-2 and JAK/STAT signals are also essential for T cell activation and persistence through the activation of the JAK kinase and STAT3/5 transcription factor signaling pathways. Given the increased understanding of CAR-T cells, it is known that CAR T cells have been modified to become fifth-generation CAR T cells. The fifth-generation CAR contained a TCR signal-transduction moiety, costimulatory domains (CD), an additional cytoplasmic domain derived from IL-2Rβ and a STAT3/5 binding motif, providing antigen-dependent cytokine signaling (51). The role of immunomodulatory genes, including *UNC13D, LYST, PRF1*, *DNMT3A*, etc., is increasingly being recognized. Ishii et al. reported that one patient who developed severe CRS associated with HLH following CD19 CAR therapy for ALL was found to carry a mutation in the perforin (*PRF1*) gene, which predisposes to HLH (52). The HLH-phenotype in PRF1-deficient patients included late expansion and/or persistence of activated CAR-T cells. Deleting DNMT3A in CAR T cells prevents exhaustion and enhances antitumor activity (53). Moreover, chemokines enhance tumor T cell infiltration to enable cancer immunotherapy. Finally, T-cell metabolism-related genes were included in the analysis panel.

In this study, based on previous research methods on tumor somatic mutations, we focused on germline mutations in patients (33, 54). The differential germline mutation analysis of the two groups found that the enrichment of T cell-related germline gene mutations appeared in patients with T cell defects during CAR-T therapy (**Figure 4B**). Apart from universal CAR T-cell therapy, autogenous CAR-T cells were harvested from patients’ lymphocytes for modification. T cell-related germline alterations might lead to T-cell defects, which means a virtual lack of functional T cells and immune function. Patients with T-cell PID are generally categorized into the absence of T cells, the presence of B cells (T^−^, B^+^), or the absence of both T and B cells (T^−^, B^−^). However, normal T-cell numbers do not exclude the possibility of T-cell defects. These findings suggest that further investigations of T-cell function-related PID in CAR T-cell immunotherapy are warranted.

We speculate that for patients with inborn errors of immunity, autologous CAR-T cells may have expansion and persistence barriers, weakening CAR-T cell efficacy and leading to a poor prognosis. This new mechanism complements the conventional CAR-T resistance mechanism. Germline genetic characteristics remind us to consider germline mutation screening before choosing CAR-T products. Universal CAR-T cells, or fully or half-matched CAR-T cells from healthy relatives, may give rise to improved therapeutic effects for patients with T-cell immunodeficiency. Allogeneic hematopoietic stem cell transplantation might be a curative method for PID (55). Moreover, the recurrent differential mutations between the two groups might explain the mechanism of T cell defects and provide a new insight for future CAR-T transformation. Significantly, alterations in the *UNC13D* and *CX3CR1* genes were enriched in the T cell defect group.

*UNC13D*, which encodes the Munc13-4 protein, was the most frequently differentially mutated gene between the two groups. Activation of the TCR signaling pathway induces Munc13-4 expression in CD8^+^ T cells (56). Munc13-4 expression is obligatory for exocytosis of lytic granules, facilitating cytotoxicity by T cells and NK cells. To date, all reported pathogenic *UNC13D* mutations evaluated for protein expression cause a marked reduction in munc13-4 protein expression (57). Germline mutations of *UNC13D* are associated with familial hemophagocytic lymphohistiocytosis type 3 (FHL3, MIM 608898). *UNC13D* deficiency-induced significantly less CD107a surface expression in CD8^+^T cells and NK cells, resulting in T cell dysfunction in degranulation (58). Lack of cytotoxicity and antigen stimulation may be responsible for CAR-T cell defects in therapy.

The compound heterozygous *CX3CR*1^I249/M280^ variant had specific intergroup differences, which led to the suppression of CX3CR1 protein expression. Both missenses were defined as pathogenic and risk factors by the ClinVar database. In addition, various studies indicated that in CX3CR1-deficient CD8^+^T cells, the coinhibitory tumor receptors such as PD-1, TIM3, LAG3, and TIGIT exhibited significantly lower levels, production of effector cytokines such as IL-2 demonstrated significantly higher levels, and they also exhibited substantially lower cytotoxicity than their CX3CR1-high counterparts did both *in vivo* and *in vitro* (59, 60). The specific high expression of the chemokine CX3CL1 in DLBCL was revealed by The Cancer Genome Atlas data, which provided a solid foundation for increasing the homing ability of CX3CR1^+^ cells. Moreover, recent studies revealed that CX3CR1^+^CD8^+^T cell subsets not only precisely predicted early response in anti-PD1 therapy, but also enhanced the anti-tumor efficacy *in vitro* (60, 61). These results strongly suggest that the deficiency of CX3CR1 targeted on the CX3CR1/CX3CL1 axis may impair the CAR-T therapeutic effect by inducing immune cell infiltration and CAR-T cell homing in DLBCL. Furthermore, more works are needed in the future to explore the underlying mechanism and to ultimately improve the curative effect of immunotherapies for lymphoma.

Notably, though limited by sample size and the single-center nature of our high-throughput sequencing study, the current study lacks external data to support our theories further. However, we aimed to validate our model in a larger-scale multicentered study in future explorations. Considering the PID genetic diversities among different human races, we believe future research including multiple populations would provide more consolidated evidence. Further validation of these new findings and frequently mutated genes (e.g., *UNC13D*, *CX3CR1*) is helpful for determining the pathogenesis of T cell dysfunction and developing novel therapeutic strategies for CAR modification in r/r DLBCL.

The results of our studies suggest that, in CD19 CAR T-cell therapy, targeted characteristics in r/r DLBCL could be used to evaluate the prognosis of T cell dysfunction related primary resistance. First, higher LDH before leukapheresis is correlated with poorer T-cell functionality. Freezing hemopoietic stem cells in the state of low LDH burden will benefit patients. Second, those who experienced high-grade CRS were more likely to have more significant CAR transgene expansion and better T-cell functionality. Third, inborn immunity errors of polygenic heterozygous variants (e.g., T-cell signaling, T-cell cytotoxicity, T-cell regulation) potentially offer clinically meaningful strata for the early identification of high-risk individuals. Allogeneic or universal CAR-T products might be an optimal treatment and overcome this situation.

In summary, our analysis builds on the clinical examination of primary resistance in cellular immunotherapy by the addition of a T-cell-related germline genetic nosology that may inform resistance mechanisms. Our investigation revealed a new interrelationship between pathogenic germline alterations and the dynamic characteristics of the CAR transgene. This work may help explain the underlying mechanism of primary resistance to treatment and provide novel insights into CAR T-cell immunotherapy.

## Data Availability Statement

The datasets presented in this study can be found in online repositories. The names of the repository/repositories and accession number(s) can be found below: NCBI, BioProject: PRJNA804958, https://dataview.ncbi.nlm.nih.gov/.

## Ethics Statement

This study was carried out following the Declaration of Helsinki and approved by the Medical Ethics Committee of the Department of Hematology, Tongji Hospital, Tongji Medical College, Huazhong University of Science and Technology (ChiCTR-OPN-16009847, ChiCTR-OPN-16008526). The ethics committee waived the requirement of written informed consent for participation.

## Author Contributions

JW analyzed the data and wrote the manuscript. KS and WM analyzed the data. WL, MZ, WZ, and ZL revised the manuscript and were in charge of the manuscript’s final approval. TG, ZZ, SZ, CC, SX, LZ, and LC performed the experiments. NW and LH provided clinical information. DL, MX, and JZ directed the research. All authors contributed to the article and approved the submitted version.

## Funding

This work was supported by the National Natural Science Foundation of China (No. 81770211 to MX) and the National Natural Science Foundation of China (No. 81630006 and No.81830008 to JZ).

## Conflict of Interest

Author SZ was employed by Wuhan Bio-Raid Biotechnology Co., Ltd.

The remaining authors declare that the research was conducted in the absence of any commercial or financial relationships that could be construed as a potential conflict of interest.

## Publisher’s Note

All claims expressed in this article are solely those of the authors and do not necessarily represent those of their affiliated organizations, or those of the publisher, the editors and the reviewers. Any product that may be evaluated in this article, or claim that may be made by its manufacturer, is not guaranteed or endorsed by the publisher.

## Supplementary Material

Supplementary Figure 1 Two clinical trials (Trial A and Trial B) were included in the analysis. Trial A involves a murine CAR19/22 T cell “cocktail” therapy, and Trial B involves an ASCT followed by CAR19/2 T cell “cocktail” therapy. Timeline of leukapheresis, leukodepletion, chimeric antigen receptor therapy T-cell (CAR-T) infusion, and follow-up period. CAR-T therapy involves separating a patient’s T cells *via* apheresis and then genetically engineering the cells to produce receptors on their surfaces, called CARs. CARs are fusion proteins of an antigen-binding domain from a monoclonal antibody and one or more T-cell receptors. T cell counts are expanded to hundreds of millions, after which the cells are then infused back into the patient, selectively destroying chemotherapy-resistant cancer cells. Before CAR-T infusion, patients in Trial A received lymphodepleting chemotherapy in the form of cyclophosphamide and fludarabine (usually 2-4 days before CAR-T therapy), and the patients in Trial B were given a standard dose of the BEAM regimen (300 mg/m^2^ bis-carmustine, −6 days; 200 mg/m^2^ etoposide, −5 to −2 days; 400mg/m^2^ cytarabine, −5 to −2days; and 140mg/m^2^ melphalan, −1 day) as myeloablative chemotherapy, which promotes *in vivo* expansion of CAR-T cells and improves their efficacy. ASCT, autologous hematopoietic stem cell transplantation; CAR, chimeric antigen receptor (CAR)-T cell (CAR-T) therapy and its complications.

Supplementary Figure 2 Study strategies of germline variants and somatic mutations. WES, whole-exome sequencing; VAF, variant allele frequency; MAF, minor allele frequency; 1000G_EAS,1000 Genome Project_East Asian; ExAC_EAS, Exome Aggregation Consortium_East Asian; gnomeAD_EAS, genome Aggregation Database_East Asian; dbsnp142, the database of SNP human build 14.

Supplementary Figure 3 Somatic clonal evolution of three patients in the T-defect group. Schematic models of evolutionary progression before and after CAR T cell infusion in three patients in the T-defect group. Primary dominant clones, secondary dominant clones, and subclones are represented in blue, red, and yellow shapes. References and VAFs of germline and somatic mutations investigated by NGS. NGS, next-generation sequencing; VAFs, variant allele frequency.

## Abbreviations

95%CI, 95% confidence interval; AE, adverse events; ASCT, autologous stem cell transplantation; B-ALL, B-cell precursor acute lymphoblastic leukemia; BEAM, bis-carmustine, etoposide, cytarabine, melphalan; BCA, B-cell aplasia; CAR, chimeric antigen receptor; C_max_, maximal expansion of transgene; COO, cell of origin; CR, complete remission; CRS, cytokine release syndrome; CTCAE, Common Terminology Criteria for Adverse Events; ddPCR, droplet digital PCR; DLBCL, diffuse large B-cell lymphoma; FISH, fluorescence *in situ* hybridization; GO, gene ontology; HLH, hemophagocytic lymphohistiocytosis; HR, hazard ratio; IL, interleukin; IPI, international prognostic index; IQRs, interquartile ranges; IUIS, International Union of Immunological Societies; KEGG, kyoto encyclopedia of genes and genomes; LDH, lactate dehydrogenase; MAF, minor allele frequency; MTD, maximal tumor diameter; NGS, next-generation sequencing; NHL, non-Hodgkin Lymphoma; NK, natural killer; NR, no response; OS, overall survival; PBMCs, peripheral blood mononuclear cells; PD, progressive disease; PET/CT, positron emission tomography/computed tomography; PFS, progression-free survival; PIDs, primary immunodeficiencies PR, partial remission; r/r, relapsed/refractory; SD, stable disease; SNPs, single-nucleotide polymorphisms; TCR, T cell receptors; T_last_, persistence of CAR transgene; TME, tumor microenvironment; ULN, upper limit of normal; WES, whole exon sequencing; WT, wild type.
