# Supplementary figures and images for: T Cell Defects: New Insights Into the Primary Resistance Factor to CD19/CD22 Cocktail CAR T-Cell Immunotherapy in Diffuse Large B-Cell Lymphoma
Source: Front Immunol. 2022 Apr 27;13:873789. doi: 10.3389/fimmu.2022.873789 (PMC9094425; doi:10.3389/fimmu.2022.873789)

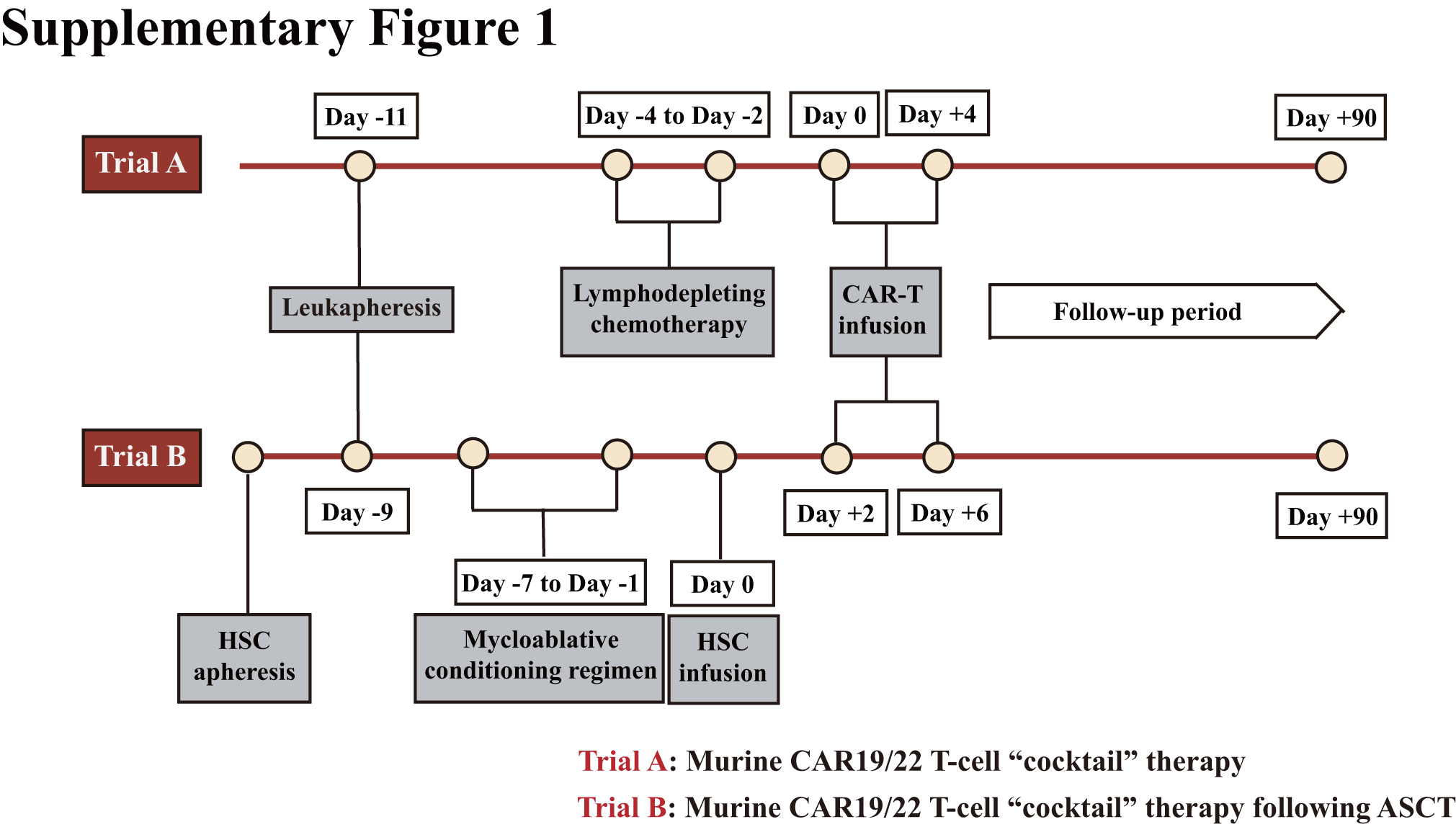

Supplement: Supplementary Figure 1 — Two clinical trials (Trial A and Trial B) were included in the analysis. Trial A involves a murine CAR19/22 T cell “cocktail” therapy, and Trial B involves an ASCT followed by CAR19/2 T cell “cocktail” therapy. Timeline of leukapheresis, leukodepletion, chimeric antigen receptor therapy T-cell (CAR-T) infusion, and follow-up period. CAR-T therapy involves separating a patient’s T cells via apheresis and then genetically engineering the cells to produce receptors on their surfaces, called CARs. CARs are fusion proteins of an antigen-binding domain from a monoclonal antibody and one or more T-cell receptors. T cell counts are expanded to hundreds of millions, after which the cells are then infused back into the patient, selectively destroying chemotherapy-resistant cancer cells. Before CAR-T infusion, patients in Trial A received lymphodepleting chemotherapy in the form of cyclophosphamide and fludarabine (usually 2-4 days before CAR-T therapy), and the patients in Trial B were given a standard dose of the BEAM regimen (300 mg/m2 bis-carmustine, −6 days; 200 mg/m2 etoposide, −5 to −2 days; 400mg/m2 cytarabine, −5 to −2days; and 140mg/m2 melphalan, −1 day) as myeloablative chemotherapy, which promotes in vivo expansion of CAR-T cells and improves their efficacy. ASCT, autologous hematopoietic stem cell transplantation; CAR, chimeric antigen receptor (CAR)-T cell (CAR-T) therapy and its complications. [file Image_1.tif]

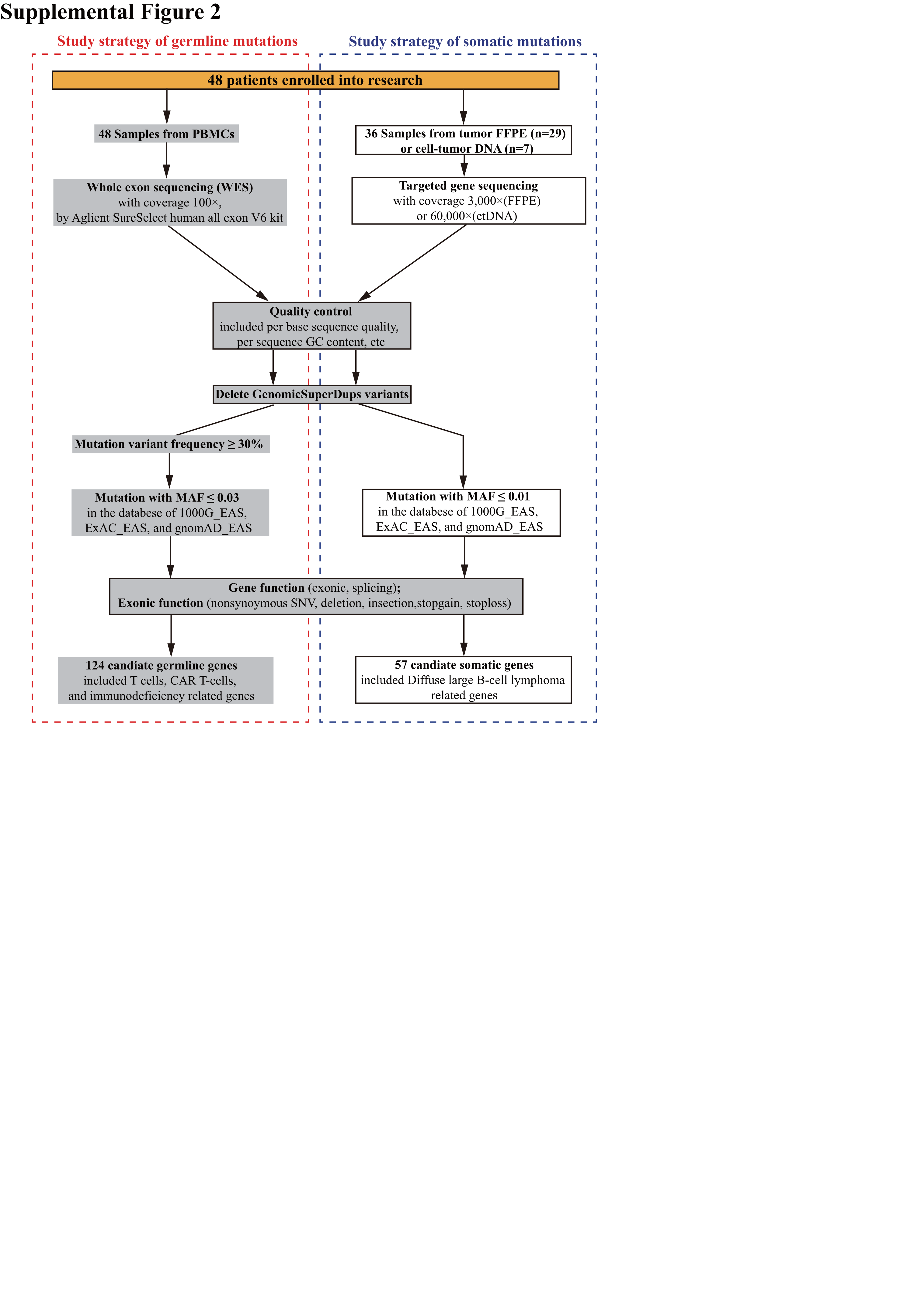

Supplement: Supplementary Figure 2 — Study strategies of germline variants and somatic mutations. WES, whole-exome sequencing; VAF, variant allele frequency; MAF, minor allele frequency; 1000G_EAS,1000 Genome Project_East Asian; ExAC_EAS, Exome Aggregation Consortium_East Asian; gnomeAD_EAS, genome Aggregation Database_East Asian; dbsnp142, the database of SNP human build 14. [file Image_2.tif]

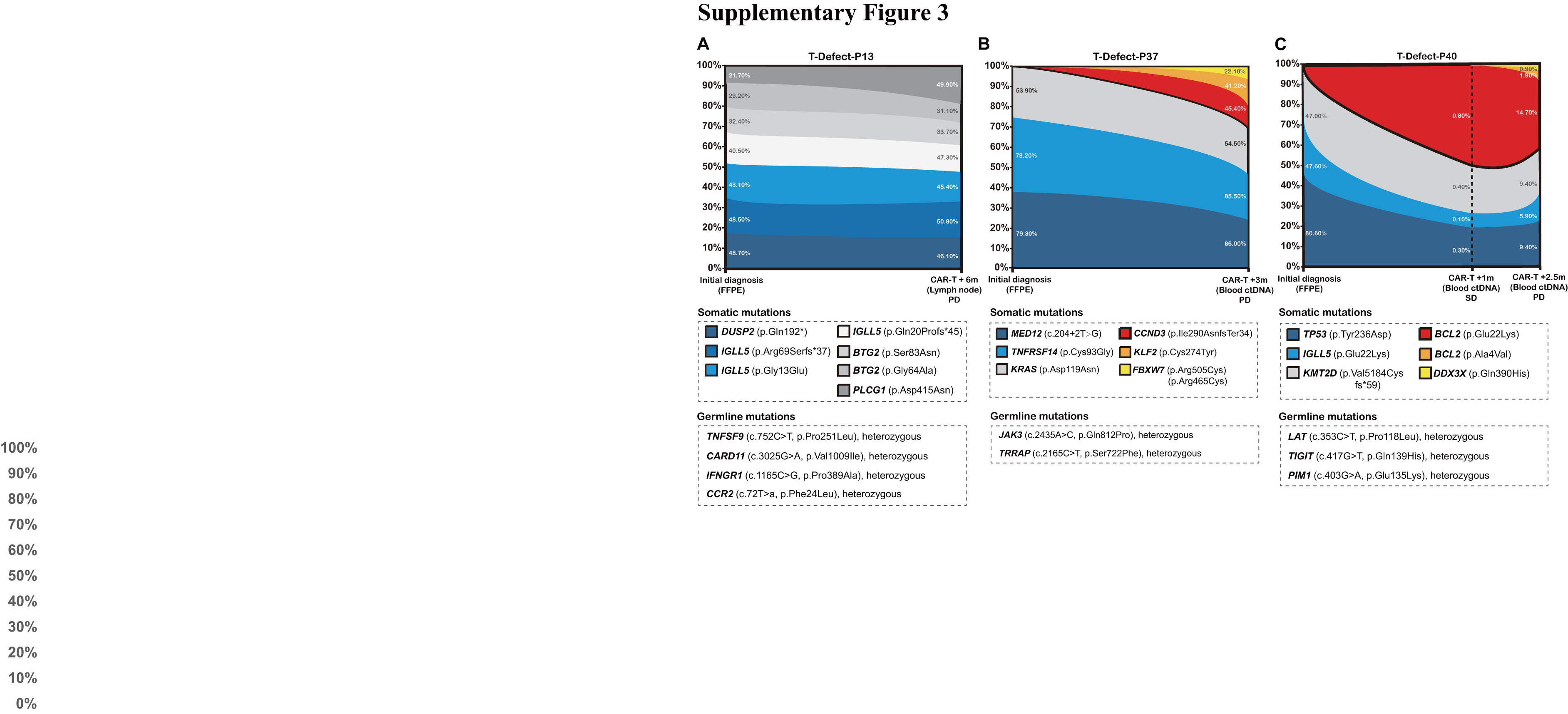

Supplement: Supplementary Figure 3 — Somatic clonal evolution of three patients in the T-defect group. Schematic models of evolutionary progression before and after CAR T cell infusion in three patients in the T-defect group. Primary dominant clones, secondary dominant clones, and subclones are represented in blue, red, and yellow shapes. References and VAFs of germline and somatic mutations investigated by NGS. NGS, next-generation sequencing; VAFs, variant allele frequency. [file Image_3.tif]
