# Supplementary material for: T Cell Defects: New Insights Into the Primary Resistance Factor to CD19/CD22 Cocktail CAR T-Cell Immunotherapy in Diffuse Large B-Cell Lymphoma
Source: Front Immunol. 2022 Apr 27;13:873789. doi: 10.3389/fimmu.2022.873789 (PMC9094425; doi:10.3389/fimmu.2022.873789)
Supplement: Supplementary file 4 [file Table_1.docx]

**Supplementary Method**

**Patient Enrollment**

A series of screening conditions were set up to select patients with typical T-cell characteristics (**Figure 2**). Firstly, a total of 223 patients with B-cell hematologic malignancies who received murine CAR-T therapy from January 2019 to August 2020 were screened at Tongji Hospital retrospectively. Secondly, to ensure that the patient's disease baseline is consistent, patients suffered from initially diagnosed diseases other than DLBCL (B-ALL, Burkitt lymphoma, Mantle cell lymphoma, Primary mediastinal B-cell lymphoma, Follicular cell lymphoma, Primary central nervous system lymphoma, B-cell lymphoblastic lymphoma)were excluded. Thirdly, patients who suffered from severe toxicity or severe adverse events were excluded, including severe infection, CRS grade 4 (Lee Criteria)(1), and grade 3 to 4 of CTCAE (v4.03 criteria). Fourthly, patients who were lost of follow-up within three months were excluded. Finally, 48 patients with DLBCL who received murine CAR-T therapy were enrolled in the research. They were divided into two groups based on the following three criteriums: (1) Expansion (Cmax ≥10,000 CAR transgene copies/μg genomic DNA) and persistence (Tlast ≥ three months) of CAR transgene detected by ddPCR, and satisfy at least one of the following rules: (2) B cell aplasia (CD19^+^B cell level/ PBMC＜1%) within three months by FC *AND/OR* (3) Remission (CR/PR) at initial efficacy assessment after three months by PET/CT scan and was divided into 2 cohorts: patients of T-strong group (n=22) vs. T-defect group (n=26). The raw data was shown in **Supplementary Table 1**.

**Whole Exon Sequencing for Germline Alterations**

The cohort consisted of 48 unrelated patients collected from Tongji Hospital, Tongji Medical College, Huazhong University of Science and Technology.

Genomic DNA extracted from peripheral blood for each sample was fragmented to an average size of 180~280bp and subjected to DNA library creation using established Illumina paired-end protocols. The Agilent SureSelect Human All ExonV6 Kit (Agilent Technologies, Santa Clara, CA, USA ) was used for exome capture according to the manufacturer’s instructions. The Illumina Novaseq 6000 platform (Illumina Inc., San Diego, CA, USA) was utilized for genomic DNA sequencing in Novogene Bioinformatics Technology Co., Ltd (Beijing, China) to generate 150-bp paired-end reads with a minimum coverage of 10× for ~99% of the genome (mean coverage of 100×).

After sequencing, basecall files conversion and demultiplexing were performed with bcl2fastq software (Illumina). The resulting fastq data were submitted to in-house quality control software for removing low quality reads, and then were aligned to the reference human genome (hs37d5) using the Burrows-Wheeler Aligner (bwa)(2), and duplicate reads were marked using sambamba tools(3).

Single nucleotide variants (SNVs) and indels were called with samtools to generate gVCF.(4) The raw calls of SNVs and INDELs were further filtered with the following inclusion thresholds: 1) read depth > 4; 2) Root-Mean-Square mapping quality of covering reads > 30; 3) the variant quality score > 20. Annotation was performed using ANNOVAR (2017June8).(5) Annotations included minor allele frequencies from public control data sets as well as deleteriousness and conservation scores enabling further filtering and assessment of the likely pathogenicity of variants.

Filtering of variants was performed as follows: 1) Delete Genomic Super duplications variants; 2) Mutations variant frequency ≥30%; 3) Variants with a MAF less than 0.03 in 1000G_EAS data(6), gnomAD_EAS data(7), and ExAC_EAS data; 4) Only SNVs occurring in exons or splice sites (splicing junction 10 bp) are further analyzed since we are interested in amino acid changes; 5) Then synonymous SNVs which are not relevant to the amino acid alternation predicted by dbscSNV are discarded; The small fragment non-frameshift (<10bp) indel in the repeat region defined by RepeatMasker are discarded. 6) Variations are screened according to scores of SIFT(8), Polyphen(9), and CADD(10) softwares. The potentially deleterious variations are reserved if the score of more than half of these three softwares support harmfulness of variations(11). Sites(>2bp) did not affect alternative splicing were removed.

**Targeted high-throughput sequencing for Somatic Alterations**

For somatic alterations, A total of 57 genes were selected in this study (Supplementary Table 2) as previous reported (12). Most genes were frequently altered in DLBCL according to data from several previously published large-scale DLBCL cohort studies (13-15). Using genome build hg19/GRCh37 as a reference, a sequencing panel covering the coding sequences (CDS) within 5 intronic base pairs around exons in 57 genes was designed online (Designstudio Sequencing, Illumina, San Diego, USA). Sequencing libraries were prepared with AmpliSeq™ Library PLUS for Illumina, using 20 ng of input genomic DNA per sample. Library sequencing was performed to 2000× coverage on a NextSeq™ 550 system using an Illumina NextSeq™ 500/550 High Output v2 Kit (Illumina, San Diego, USA). The alignment and variant calling were performed using a DNA Amplicon workflow with default parameters on BaseSpace Sequence Hub (Illumina). Generated variants were further annotated using Annovar (5).

Variant filtering was performed by the following cascade of steps: 1) select exon nonsynonymous or splice donor/acceptor site variants; 2) exclude variants with population frequency > 0.0001 in the gnomAD database unless variant is included as a somatic variant of lymphoid neoplasm in the COSMIC database; 3) exclude variants present in an in-house curated blacklist. The formation of our variant screening blacklist was based on the idea described previously by Schmitz et al (14). As these false positive variants were presumed to be artifacts generated either by the high throughput sequencing platform itself or due to errors in alignment or annotation of the sequencing reads by the analytical. pipeline. Typically, these variants were abnormally prevalent, identified exclusively in specific sequencing platform, and are not recurrent variants included in the major public cancer somatic mutation database (COSMIC database, <https://cancer.sanger.ac.uk/cosmic>). Therefore, as such variants were unique in our center and there were no universal criteria for identification, the blacklist was built for future rapid and accurate variants’ screening; 4) exclude variants found in regions with poor coverage; and 5) exclude variants with quality less than 30 or read depth less than 20. For activation-induced cytidine deaminase (AID) somatic hypermutation (SHM) analysis, we additionally selected synonymous variants and variants in intron/UTR regions, and each variant also needed to fulfill the aforementioned criteria from step 2 to step 5 (16).

**Reference**

1. Lee DW, Gardner R, Porter DL, Louis CU, Ahmed N, Jensen M, et al. Current concepts in the diagnosis and management of cytokine release syndrome. *Blood* (2014) 124(2):188-95. Epub 2014/05/31. doi: 10.1182/blood-2014-05-552729. PubMed PMID: 24876563; PubMed Central PMCID: PMCPMC4093680.

2. Li H, Durbin R. Fast and accurate short read alignment with Burrows-Wheeler transform. *Bioinformatics* (2009) 25(14):1754-60. Epub 2009/05/20. doi: 10.1093/bioinformatics/btp324. PubMed PMID: 19451168; PubMed Central PMCID: PMCPMC2705234.

3. Tarasov A, Vilella AJ, Cuppen E, Nijman IJ, Prins P. Sambamba: fast processing of NGS alignment formats. *Bioinformatics* (2015) 31(12):2032-4. Epub 2015/02/24. doi: 10.1093/bioinformatics/btv098. PubMed PMID: 25697820; PubMed Central PMCID: PMCPMC4765878.

4. Li H, Handsaker B, Wysoker A, Fennell T, Ruan J, Homer N, et al. The Sequence Alignment/Map format and SAMtools. *Bioinformatics* (2009) 25(16):2078-9. Epub 2009/06/10. doi: 10.1093/bioinformatics/btp352. PubMed PMID: 19505943; PubMed Central PMCID: PMCPMC2723002.

5. Wang K, Li M, Hakonarson H. ANNOVAR: functional annotation of genetic variants from high-throughput sequencing data. *Nucleic Acids Res* (2010) 38(16):e164. Epub 2010/07/06. doi: 10.1093/nar/gkq603. PubMed PMID: 20601685; PubMed Central PMCID: PMCPMC2938201.

6. Clarke L, Zheng-Bradley X, Smith R, Kulesha E, Xiao C, Toneva I, et al. The 1000 Genomes Project: data management and community access. *Nat Methods* (2012) 9(5):459-62. Epub 2012/05/01. doi: 10.1038/nmeth.1974. PubMed PMID: 22543379; PubMed Central PMCID: PMCPMC3340611.

7. gnomAD. Available at: <https://doi.org/10.1101/030338>. Accessed February/21, 2017.

8. Kumar P, Henikoff S, Ng PC. Predicting the effects of coding non-synonymous variants on protein function using the SIFT algorithm. *Nat Protoc* (2009) 4(7):1073-81. Epub 2009/06/30. doi: 10.1038/nprot.2009.86. PubMed PMID: 19561590.

9. Adzhubei IA, Schmidt S, Peshkin L, Ramensky VE, Gerasimova A, Bork P, et al. A method and server for predicting damaging missense mutations. *Nat Methods* (2010) 7(4):248-9. Epub 2010/04/01. doi: 10.1038/nmeth0410-248. PubMed PMID: 20354512; PubMed Central PMCID: PMCPMC2855889.

10. Kircher M, Witten DM, Jain P, O'Roak BJ, Cooper GM, Shendure J. A general framework for estimating the relative pathogenicity of human genetic variants. *Nat Genet* (2014) 46(3):310-5. Epub 2014/02/04. doi: 10.1038/ng.2892. PubMed PMID: 24487276; PubMed Central PMCID: PMCPMC3992975.

11. Muona M, Berkovic SF, Dibbens LM, Oliver KL, Maljevic S, Bayly MA, et al. A recurrent de novo mutation in KCNC1 causes progressive myoclonus epilepsy. *Nat Genet* (2015) 47(1):39-46. Epub 2014/11/18. doi: 10.1038/ng.3144. PubMed PMID: 25401298; PubMed Central PMCID: PMCPMC4281260.

12. Zhang W, Yang L, Guan YQ, Shen KF, Zhang ML, Cai HD, et al. Novel bioinformatic classification system for genetic signatures identification in diffuse large B-cell lymphoma. *BMC Cancer* (2020) 20(1):714. Epub 2020/08/02. doi: 10.1186/s12885-020-07198-1. PubMed PMID: 32736575; PubMed Central PMCID: PMCPMC7393908.

13. Reddy A, Zhang J, Davis NS, Moffitt AB, Love CL, Waldrop A, et al. Genetic and Functional Drivers of Diffuse Large B Cell Lymphoma. *Cell* (2017) 171(2):481-94 e15. Epub 2017/10/07. doi: 10.1016/j.cell.2017.09.027. PubMed PMID: 28985567; PubMed Central PMCID: PMCPMC5659841.

14. Schmitz R, Wright GW, Huang DW, Johnson CA, Phelan JD, Wang JQ, et al. Genetics and Pathogenesis of Diffuse Large B-Cell Lymphoma. *N Engl J Med* (2018) 378(15):1396-407. Epub 2018/04/12. doi: 10.1056/NEJMoa1801445. PubMed PMID: 29641966; PubMed Central PMCID: PMCPMC6010183.

15. Chapuy B, Stewart C, Dunford AJ, Kim J, Kamburov A, Redd RA, et al. Molecular subtypes of diffuse large B cell lymphoma are associated with distinct pathogenic mechanisms and outcomes. *Nat Med* (2018) 24(5):679-90. Epub 2018/05/02. doi: 10.1038/s41591-018-0016-8. PubMed PMID: 29713087; PubMed Central PMCID: PMCPMC6613387.

16. Khodabakhshi AH, Morin RD, Fejes AP, Mungall AJ, Mungall KL, Bolger-Munro M, et al. Recurrent targets of aberrant somatic hypermutation in lymphoma. *Oncotarget* (2012) 3(11):1308-19. Epub 2012/11/08. doi: 10.18632/oncotarget.653. PubMed PMID: 23131835; PubMed Central PMCID: PMCPMC3717795.
